# Novel Anti-Melanogenesis Properties of Polydeoxyribonucleotide, a Popular Wound Healing Booster

**DOI:** 10.3390/ijms17091448

**Published:** 2016-09-01

**Authors:** Tai Kyung Noh, Bo Young Chung, Su Yeon Kim, Mi Hye Lee, Moon Jung Kim, Choon Shik Youn, Mi Woo Lee, Sung Eun Chang

**Affiliations:** 1 Department of Dermatology, Asan Medical Center, University of Ulsan College of Medicine, Seoul 05505, Korea; medsnutk@gmail.com (T.K.N.); u2u2star@gmail.com (S.Y.K.); algp_@naver.com (M.H.L.); miumiu@amc.seoul.kr (M.W.L.); 2 Department of Dermatology, College of medicine, Hallym University Kangnam Sacred Heart Hospital, Seoul 07441, Korea; victoryby777@gmail.com; 3 MJ All Skin Clinic, Seoul 04537, Korea; mjallskin@gmail.com; 4 YeMiWon Clinic, Seoul 06280, Korea; dermacsyoun@hanmail.net

**Keywords:** polydeoxyribonucleotide, melanogenesis, coculture, hyperpigmentation

## Abstract

Polydeoxyribonucleotide (PDRN), a deoxyribonucleotide polymer, is popularly used for faster healing of cutaneous wounds and boosting of neocollagenesis of photoaged skin among current dermatologic practitioners. Some patients receiving PDRN injection treatment also reported improvement of photoaging-associated mottled pigmentation (PMP). To investigate the effect of PDRN on cutaneous melanogenesis, we examined the effect of PDRN and an available product (Placentex^®^) containing PDRN on melanogenesis using human melanocytes-keratinocytes cocultures and mouse melanocytes. Melanin content, tyrosinase activity, and levels of microphthalmia-associated transcription factor (MITF), tyrosinase, and tyrosinase-related protein (TRP-1) were determined. Intracellular signaling pathways were assessed by Western blotting. PDRN and Placentex^®^ led to decreases in melanin content, tyrosinase activity, and MITF and TRP-1 expression with concomitant increases in phosphorylated forms of extracellular signal-regulated protein kinase (ERK) and AKT in mouse melanocytes. More importantly, both PDRN and Placentex^®^ significantly suppressed the melanin content in human melanocyte–keratinocyte cocultures. Clinical evaluation of six female patients with facial hyperpigmentation after three sessions of intradermal PDRN injections using a 5-point scale revealed that PDRN led to more than noticeable improvements in hyperpigmented lesions. This is the first study to demonstrate that PDRN, which is known for its wound-healing properties, may have novel anti-melanogenesis and potential skin whitening properties.

## 1. Introduction

Polydeoxyribonucleotide (PDRN), a wound healing booster that has recently gained popularity in dermatological practice in a number of countries including Korea, is extracted from the sperm of trout and contains deoxyribonucleotide polymers of 50–2000 base pairs. PDRN was shown to be effective for the treatment of a wide range of disorders such as diabetic foot ulcers, scars, vascular insufficiency, and female pattern hair loss [1,2,3]. Although precise mechanism of action of PDRN is not known, it is used as a wound healing and anti-dystrophic agent with anti-inflammatory properties acting via reduction in cytokine levels [1,2,3,4]. PDRN was shown to exert its effects via activation of adenosine A_2A_ receptors that regulate the cytokine network by inhibiting the secretion of inflammatory cytokines from macrophages in vitro [2,4,5]. The association between adenosine A_2A_ receptors and PDRN in inflammation was illustrated by the anti-inflammatory effect of topical PDRN gel application in periodontitis [6] and abrogation of PDRN-mediated anti-inflammatory effects by specific A_2A_ antagonists [5,6]. A recent study suggested that high-dose adenosine might inhibit pigmentation through negative regulation of tyrosinase [7], which activated adenosine A_2__A_ receptor and the salvage pathway, leading to induced secretion of growth factors including vascular endothelial growth factor (VEGF) and anti-inflammatory cytokines [3,5,6].

Asian skin is prone to post-inflammatory hyperpigmentation (PIH) after laser skin resurfacing and peels [8]. PDRN has been utilized for its ability to enhance wound healing; thus, PDRN as posttreatment is expected to decrease PIH sequelae. Despite its popular use for treatment and prevention of hyperpigmentation, the mechanism underlying anti-melanogenesis properties of PDRN is unknown. Intradermal PDRN injection is increasingly used in dermatological practice for Asian facial skin in a number of conditions including laser toning-resistant photoaging-associated mottled pigmentation (PMP) due to chronic ultraviolet (UV) radiation, melasma with inflammatory features, and PIH due to laser treatment. Thus, we investigated the effects of PDRN on melanogenesis in a coculture model of human melanocytes and keratinocytes as well as in melanocytes.

## 2. Results

### 2.1. Polydeoxyribonucleotide and Placentex^®^ Inhibit Melanogenesis in Mel-Ab Cells and in Human Melanocyte–Keratinocyte Cocultures

**Figure 1:**
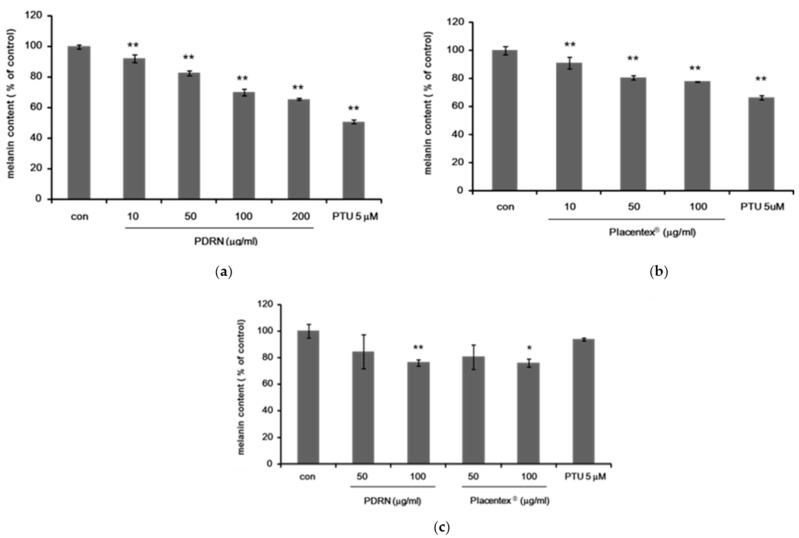
Polydeoxyribonucleotide and Placentex^®^ inhibit melanogenesis in Mel-Ab cells and in human melanocyte–keratinocyte cocultures. (**a**) Polydeoxyribonucleotide (PDRN) inhibits melanogenesis in Mel-Ab cells. Melanin content of Mel-Ab cells treated with PDRN (10–200 μg/mL) for 4 days. *N*′-phenylthiourea (PTU, 5 μM) was used as a positive control. Data represent mean ± standard deviation (SD) of triplicate assays expressed as percentage of control. * *p* < 0.05, ** *p* < 0.01 compared to untreated control; (**b**) Placentex^®^ inhibits melanogenesis in Mel-Ab cells. Melanin content of Mel-Ab cells treated with Placentex^®^ (10–100 μg/mL) for 4 days. PTU (5 μM) was used as a positive control. Data represent mean ± SD of triplicate assays expressed as percentage of control. * *p* < 0.05, ** *p* < 0.01 compared to untreated control; (**c**) PDRN and Placentex^®^ inhibit melanogenesis in normal human melanocytes cocultured with human keratinocytes. Melanin content of human melanocytes treated with PDRN (50–100 μg/mL) or Placentex^®^ (50–100 μg/mL) for 5 days. PTU (5 μM) was used as a positive control. Data represent mean ± SD of triplicate assays expressed as percentage of control. * *p* < 0.05, ** *p* < 0.01 compared to untreated control.

To determine whether PDRN and Placentex^®^ inhibited melanogenesis, we first examined the reduction of melanin synthesis in an immortal murine melanocyte cell line, Mel-Ab. Melanin content of Mel-Ab cells treated with PDRN (10–200 μg/mL) or Placentex^®^ (10–100 μg/mL) for 4 days was measured, and *N′*-phenylthiourea (PTU, 5 μM) was used as a positive control (Figure 1a,b).

Functional epidermal units are formed by one melanocyte surrounded by about 10 keratinocytes. Regulation of skin pigmentation is a complicated process involving interactions among melanocytes, keratinocytes, and fibroblasts facilitated via direct cell–cell contact and various paracrine mechanisms. Keratinocytes secrete several melanogenic and/or proliferating factors recognized by corresponding receptors on melanocytes. As several studies demonstrated that PDRN facilitated wound healing, the anti-melanogenesis effects of PDRN were assessed using cocultures of human melanocytes and keratinocytes due the cell–cell interaction that is necessary for PCRN to exert its effects. We hypothesized that cocultures of human melanocytes and keratinocytes would be an appropriate mode to investigate the potential anti-melanogenesis effects of PDRN. Thus, we next determined the melanin content of human melanocyte–keratinocyte cocultures treated with PDRN (50–100 μg/mL) or Placentex^®^ (50–100 μg/mL) for 5 days. We found that there was a significant inhibition of melanin synthesis by PDRN and Placentex^®^ in a dose-dependent manner (Figure 1c).

### 2.2. Polydeoxyribonucleotide and Placentex^®^ Suppress Intracellular Tyrosinase Activity in Mel-Ab Cells

**Figure 2:**
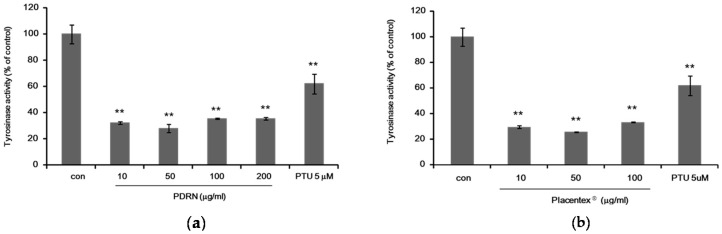
Polydeoxyribonucleotide and Placentex^®^ suppress intracellular tyrosinase activity in Mel-Ab cells. (**a**) Polydeoxyribonucleotide (PDRN) suppresses tyrosinase activity in Mel-Ab cells. Mel-Ab cells were incubated with 10–200 μg/mL PDRN for 4 days, and cellular tyrosinase activity was measured. *N′*-phenylthiourea (PTU, 5 μM) was used as a positive control. Data represent mean ± standard deviation (SD) of triplicate assays expressed as percentage of control. * *p* < 0.05, ** *p* < 0.01 compared to untreated control; (**b**) Placentex^®^ represses tyrosinase activity in Mel-Ab cells. Mel-Ab cells were incubated with 10–100 μg/mL Placentex^®^ for 4 days, and cellular tyrosinase activity was measured. PTU (5 μM) was used as a positive control. Data represent mean ± SD of triplicate assays expressed as percentage of control. ** *p* < 0.01 compared to untreated control.

Tyrosinase activity in Mel-Ab cells treated with PDRN or Placentex^®^ was examined by an intracellular tyrosinase assay. Mel-Ab cells were incubated with 10–200 μg/mL of PDRN or 10–100 μg/mL of Placentex^®^ for 4 days. *N′*-phenylthiourea (PTU, 5 μM) was used as a positive control. The results showed that both PDRN and Placentex^®^ reduced intracellular tyrosinase activity in Mel-Ab cells (Figure 2a,b).

### 2.3. Polydeoxyribonucleotide and Placentex^®^ Reduce the Levels of Microphthalmia-Associated Transcription Factor and Melanogenesis-Related Proteins

**Figure 3:**
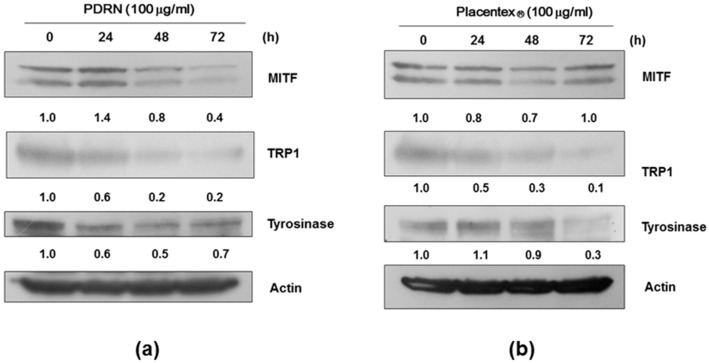
Polydeoxyribonucleotide and Placentex^®^ reduce the levels of microphthalmia-associated transcription factor and melanogenesis-related proteins in Mel-Ab cells. Whole-cell lysates were analyzed by western immunoblotting using antibodies against microphthalmia-associated transcription factor (MITF), tyrosinase-related protein (TRP)-1, and tyrosinase. Normalization was achieved by dividing the densitometric values for individual bands by the densitometric value for β-actin for the same sample. (**a**) Polydeoxyribonucleotide (PDRN) reduces the levels of melanogenesis-related proteins. Mel-Ab cells were incubated with 100 μg/mL PDRN for 24–72 h; (**b**) Placentex^®^ reduces the levels of melanogenesis-related proteins. Mel-Ab cells were incubated with 100 μg/mL Placentex^®^ for 24–48 h.

Microphthalmia-associated transcription factor (MITF) is a key transcription factor in melanogenesis that upregulates the transcription of members of the tyrosinase gene family such as tyrosinase, tyrosinase-related protein (TRP)-1, and TRP-2. Thus, we next investigated the effects of PDRN and Placentex^®^ on protein levels of MITF, tyrosinase, and TRP-1. Mel-Ab cells were treated with 100 μg/mL PDRN or Placentex^®^ for 24–72 h; whole-cell lysates were then analyzed by western immunoblotting. β-Actin was used for normalization by densitometric analysis. As shown in Figure 3a,b, the protein levels of MITF, tyrosinase, and TRP-1 were decreased in Mel-Ab cells treated with PDRN or Placentex^®^.

### 2.4. Polydeoxyribonucleotide (PDRN) Affects the Levels of Melanogenesis-Related Signaling Pathways

**Figure 4:**
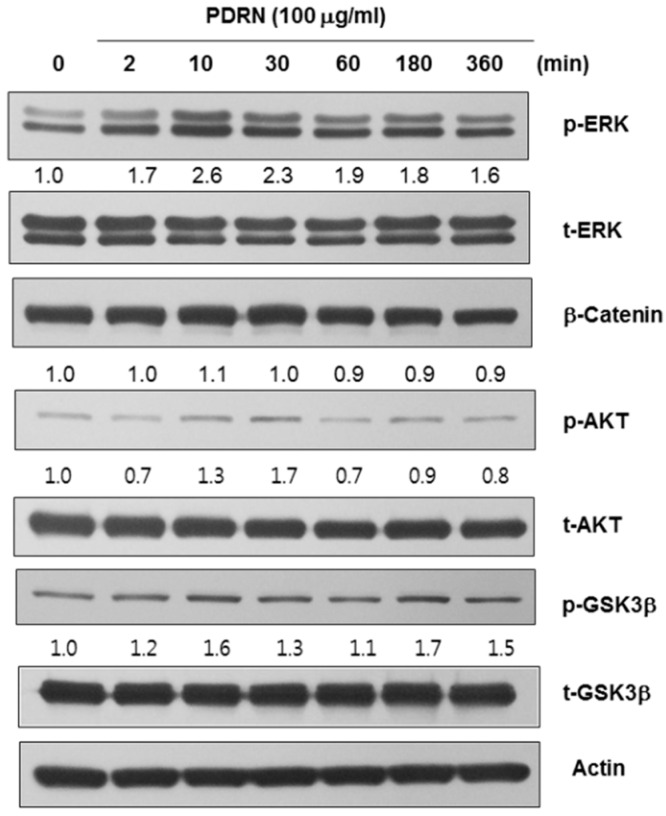
Polydeoxyribonucleotide affects the levels of melanogenesis-related signaling pathways. Polydeoxyribonucleotide (PDRN) induced the phosphorylation of extracellular signal-regulated protein kinase (ERK) and AKT within 10–30 min after treatment, whereas the levels of β-catenin or phosphorylated-GSK3β were not affected under identical conditions.

Signaling by mitogen-activated protein (MAP) kinases, including extracellular signal-regulated protein kinase (ERK), and AKT is suggested to suppress melanogenesis through the degradation of MITF. Additionally, upregulation of ERK signaling was shown to be associated with the downregulation of melanin synthesis [9,10]. Thus, to further investigate the role of PDRN in melanogenesis, we examined protein levels of melanogenesis-associated signaling molecules such as ERK, AKT, and glycogen synthase kinase 3 beta (GSK3β). As presented in Figure 4, there was a transient increase in the levels of phosphorylated ERK (p-ERK) and phosphorylated AKT (p-AKT) in Mel-Ab cells treated with PDRN for 10–30 min. Conversely, while the levels of phosphorylated-GSK3β fluctuated during 10–360 min of PDRN treatment, p-ERK and p-AKT levels steadily increased between 10 and 30 min after the addition of PDRN, with subsequent decreases after 60 min posttreatment. Finally, PDRN did not lead to any changes in the protein levels of β-catenin or phosphorylated-GSK3β.

### 2.5. Clinical Efficacy of Placentex^®^ for the Treatment of Hyperpigmentation

To confirm the anti-melanogenesis effect of PDRN, we clinically evaluated six Korean female patients with facial hyperpigmentation such as laser toning-resistant mottled pigmentation, melasma, and pigmented contact dermatitis. We performed manual intradermal injection of Placentex^®^ at a dose of 0.05–0.1 mL per 1-cm^2^ injection site. A total average of 1 mL Placentex^®^ was injected in each subject per session. Almost all hyperpigmented areas received injections at 4-week intervals for a total of three sessions. The evaluations were conducted at baseline and after 4 weeks at the conclusion of three treatment sessions. The evaluations were conducted at baseline and at 4 weeks after three treatment sessions and digital photographs obtained under identical conditions (room, light source, and camera setting) were used to document and assess patients. 

**Figure 5:**
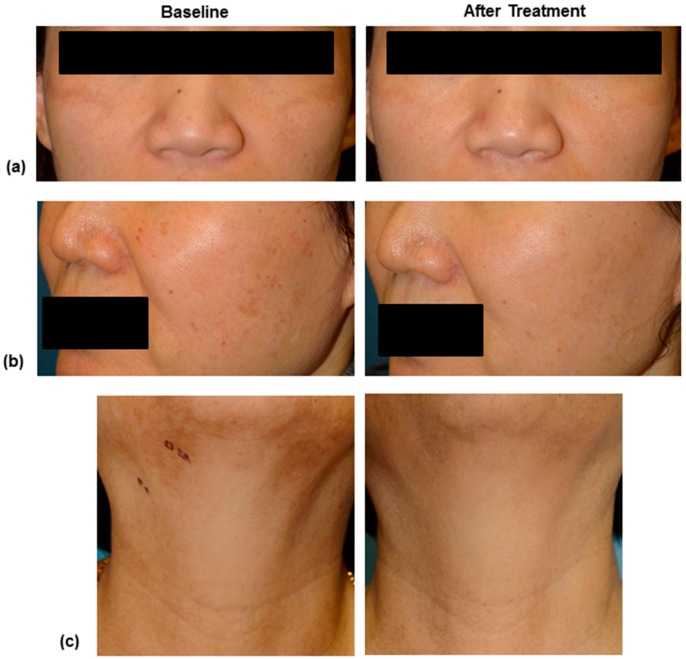
Standard digital photography images of patient number 1 (**a**), 5 (**b**) and 6 (**c**) at baseline and 4 weeks after 3 times of intradermal PDRN injection treatment. The clinical photographs of right side demonstrate significant improvement after 3 sessions of intralesional injection of Placentex^®^ treatment.

Two independent dermatologists evaluated the digital photographs of patients using a 5-point scale as follows: 1, little or no improvement (0%–10%); 2, noticeable improvement (10%–25%); 3, fair improvement (25%–50%); 4, good improvement (50%–75%); and 5, excellent improvement (>75%). As seen in Table 1 and Figure 5a–c, all patients exhibited improvement in scores by more than 2 points from baseline, using the 5-point scale. All patients completed this pilot clinical study. The mean age (±standard deviation) was 45.2 (±12.0) years with a range of 34–66 years. The Fitzpatrick skin types of patients were III (*n* = 3) and IV (*n* = 3) in this study. (Table 1 and Figure 5a–c).

## 3. Discussion

Melanin is the determinant of a person’s skin color and protects against UV radiation-induced damage. However, the overproduction and accumulation of melanin due to prolonged exposure to UV irradiation or chronic inflammation can lead to various hyperpigmentation skin disorders such as melasma, mottled hyperpigmentation, freckles, senile lentigines, and post-inflammatory hyperpigmentation. Melanin is produced by melanocytes via an enzymatic cascade that is tightly regulated by tyrosinase, TRP-1, and TRP-2 [11,12]. Tyrosinase, which converts tyrosine to dopaquinone, is the key enzyme involved in the rate-limiting step of tyrosine metabolism [13]. MITF is a central regulator of the survival and proliferation of melanocytes and promotes the transcription of the genes related to melanogenesis such as tyrosinase and TRP-1 [14]. 

PDRN is extracted from the sperm of trout (*Oncorhynchus mykiss*, (*O. mykiss*)) or salmon (*Oncorhynchus keta*, (*O. keta*)) contains deoxyribonucleotide polymers with specific molecular weight distribution [15]. PDRN was initially described as a tissue-repair stimulating agent extracted from human placenta, and molecular weight distribution of the PDRN pool in the formulation of Placentex^®^ clearly indicates that PDRN is the active component, based on the specific range of 50–2200 base pairs determined by electrophoresis and high-performance liquid chromatography [16]. Evidently, the source of PDRN is distinct between human placenta and sperm of *O. mykiss* and *O. keta*; however, previous studies clearly demonstrated that PDRN from different sources exhibited similar properties in wound healing in skin disorders and other diseases via PDRN-A_2A_ pathway [15,17]. PDRN also can trigger the salvage pathway for the synthesis of nucleic acid, nucleosides, and nucleotides. All together, these findings suggest that PDRN might play important roles in skin rejuvenation and energy saving metabolism [18]. Placentex^®^, as a natural marine product, was developed by selective extraction from trout or salmon sperm; one vial contains 5.625 mg PDRN in 3 mL as the active ingredient. Therapeutic administration of one vial PDRN includes intramuscular or subcutaneous injection in a 15–20-day cycle that can be repeated, according to physician recommendations. In vitro and in vivo studies established that PDRN promoted growth of human primary fibroblasts and that exogenous PDRN supplementation protects the repair of cyclobutane pyrimidine dimers in UVB-exposed dermal fibroblasts in a dose- and time-dependent manner [2,15]. 

Adenosine is a purine nucleoside released from a variety of cells in response to a range of stressors [19]. Adenosine exerts a variety of biological effects such as modulation of innate immunity, vascular pathologies, and hematopoiesis via purinergic receptors (A_1_, A_2A_, A_2B_, and A_3_) [20,21]. PDRN specifically acts through the activation of A_2A_ receptor, which leads to endothelial cell proliferation, migration, and secretion of VEGF [3]. VEGF was found to act as a stimulant in cell lines such as osteoblasts, fibroblasts, and pre-adipocytes [22,23]. In previous studies, PDRN was found to enhance wound healing in chronic diabetic foot ulcers and graft donor sites [2] and was shown to have anti-inflammatory effects in a mouse model of arthritis via reducing the levels of proinflammatory cytokines such as tumor necrosis factor (TNF)-α and interleukin-6 [4]. 

Recent studies found that adenosine reduced inflammation by suppressing the activity of most immune cells [19,20]. A_2A_ receptor signaling was proposed to inhibit lipopolysaccharide (LPS)-induced proinflammatory cytokine production through a unique cyclic adenosine monophosphate (AMP)-dependent pathway [5,24]. Köröskényi et al. [5] showed that loss of A_2A_ receptors in A_2A_ receptor-null macrophages did not alter LPS-induced NF-κB activation but led to enhanced basal and LPS-induced phosphorylation of MAP kinases in mouse macrophage cells. They also suggested that A_2A_ receptor signaling could regulate MAP kinases including ERK and c-Jun N-terminal kinases (JNKs), through modulation of dual specific phosphatase (DUSP) 1 expression in mouse macrophages [5].

To our knowledge, our present study is the first to show PDRN-mediated regulation of melanogenesis in vitro. In the present study, treatment of melanocytes with PDRN and PDRN containing Placentex^®^ inhibited melanogenesis. Melanogenesis is regulated by several biochemical steps, in which many melanogenesis-related enzymes are coordinately involved. Melanogenesis is regulated mainly by the activity and expression of the rate-limiting enzyme tyrosinase, a copper-containing glycoprotein. TRP-1 and TRP-2 are two other major melanogenic enzymes, whereas MITF is a major transcription factor involved in the regulation of the abovementioned melanocyte-specific enzymes [10,25,26]. In this study, PDRN treatment led to decreases in protein levels of tyrosinase, TRP-1, and MITF. We also showed that PDRN, as an activator of adenosine A_2A_ receptor led to the accumulation of p-ERK and p-AKT via Western immunoblot analysis. These findings suggested that PDRN, via activation of A_2A_ receptor, might exert its effects through the activation of the MAP kinase pathway in melanocytes, similar to that reported in mouse macrophage. Melanogenesis can be regulated by several signaling pathways including the MAP kinase pathway. Upregulation of ERK signaling was shown to downregulate melanin synthesis [9,27]. AKT is the typical effector of phosphatidylinositide 3-kinases (PI3K); inhibition of PI3K leads to increased melanin synthesis through increased transcription of tyrosinase and TRP-1 resulting from the increased expression of MITF [10]. Therefore, activation of ERK and PI3K/AKT signaling reduces melanogenesis via downregulation of MITF expression [10,28]. Moreover, a recent study showed that fast wound healing by epidermal growth factor (EGF) effectively inhibited PIH due to CO_2_ fractional laser-conditioned media containing prostaglandin E2 (PGE2) [8]. As such, PDRN, which has anti-inflammatory effects, might negatively modulate arachidonic acid pathway resulting in reduced PGE2 that is responsible for PIH. However, EGF was found not to have direct anti-melanogenesis effects in melanocytes in that study [8]. 

This study demonstrates that PDRN suppressed melanogenesis via the reduction of MITF and its downstream targets. These results reveal the underlying mechanism of action of PDRN and illustrate this as a new promising therapeutic approach for PIH in esthetic dermatology.

## 4. Materials and Methods 

### 4.1. Materials 

Dulbecco’s modified Eagle’s medium (DMEM) and fetal bovine serum (FBS) were purchased from WelGENE Inc. (Daegu, Korea). PDRN and Placentex^®^ (Mastelli, Sanremo, Italy) were obtained from Pharmaresearch Products (Seongnam, Korea). l-3,4-Dihydroxyphenylalanine (l-DOPA), Cholera Toxin (CT), phorbol 12-myristate 13-acetate (TPA), and the polyclonal antibody against actin were purchased from Sigma-Aldrich (St. Louis, MO, USA). Tyrosinase antibody (C-19) was purchased from Santa Cruz Biotechnology, Inc. (Dallas, TX, USA), MITF antibody (Ab-1) was obtained from NeoMarkers (Fremont, CA, USA), and antibody specific for TRP-1 was purchased from Abcam (Cambridge, UK). Antibodies specific for phospho-ERK1/2, total-ERK1/2, phospho-GSK3β, total-GSKβ, phospho-AKT, total-AKT, and β-catenin were purchased from Cell Signaling Technology (Beverly, MA, USA).

### 4.2. Cell Culture

Mel-Ab cell line is derived from spontaneously immortalized mouse melanocytes and synthesizes large quantities of melanin. Mel-Ab cells were maintained in DMEM supplemented with 10% FBS, 100 nM TPA, 1 nM CT, and 1% antibiotic/antimycotic solution at 37 °C in 5% CO_2_. Human neonatal epidermal melanocytes with moderate pigmentation were cultured in medium 254 supplemented with human melanocyte growth supplement (Cascade Biologics, CA, USA). Melanocytes were used between passages 3 and 7. Human neonatal keratinocytes were cultured in Epilife^®^ medium supplemented with human keratinocyte growth supplement (HKGS; Cascade Biologics, CA, USA). Keratinocytes were used between passages 2 and 5. Both melanocytes and keratinocytes were incubated at 37 °C in 5% CO_2_.

For our coculture model, melanocytes were plated on 6-well plates at a density of 6 × 10^4^ cells per well. After 24 h, keratinocytes were added to each well at a density of 3 × 10^5^ cells for cocultures, with an initial seeding ratio of 5:1. Cocultures were then maintained in keratinocyte media. Twenty-four hours later, wells were treated two times with PDRN and Placentex^®^, and melanin content was measured 5 days later.

### 4.3. Measurement of Melanin Content

Mel-Ab cells were treated with PDRN and Placentex^®^ in DMEM containing 10% FBS for 4 days. In our melanocyte-keratinocyte coculture model, cells were treated with PDRN and Placentex^®^ for 5 days. Cells were resuspended in 550 µL 1 N NaOH at 100 °C for 30 min and centrifuged at 13,000 rpm for 5 min. Optical density (OD) of the supernatants were measured at 405 nm using a microplate reader. Melanin production was expressed as percentage change from that measured in untreated controls.

### 4.4. Intracellular Tyrosinase Activity Assay 

Tyrosinase activity was determined as described previously, with slight modifications [29]. Briefly, Mel-Ab cells were seeded in 6-well plates and incubated with PDRN and Placentex^®^ for 4 days. Cells were then washed with ice-cold phosphate-buffered saline (PBS) and lysed with phosphate buffer (pH 6.8) containing 1% Triton X-100. The cells were then disrupted by a freeze/thaw cycle, and lysates were collected by centrifugation at 15,000 rpm for 10 min. After determination of protein levels, protein concentrations of all samples were equalized with lysis buffer. A total of 90 µL/sample was loaded used in each well of a 96-well plate, and 10 µL of 10 mM l-DOPA was added to all wells. Control wells contained 90 µL of lysis buffer and 10 µL of 10 mM l-DOPA. Following incubation at 37 °C, absorbance at 475 nm was measured every 10 min for at least 1 h using a microplate reader.

### 4.5. Western Immunoblotting

Cells were lysed in protein lysis buffer (Intron, Seongnam, Korea) and centrifuged at 13,000 rpm for 10 min. Protein concentrations were determined using a bicinchoninic acid protein assay kit. Next, 20 μg of protein per lane was separated by SDS-polyacrylamide gel electrophoresis and transferred to nitrocellulose membranes, which were then blocked with 5% nonfat milk in Tris-buffered saline containing 0.5% Tween 20. Blots were then incubated with the appropriate primary antibodies at a dilution of 1:1000, followed by incubation with horseradish peroxidase-conjugated secondary antibodies. Immunodetection was achieved using an enhanced chemiluminescence kit (Pierce, Rockford, IL, USA). Image analysis to determine relative band densities was performed using Image J software (http://reb.info.nih.ogv/ij/).

### 4.6. Patients and Study Design

Six Korean females (mean age, 45.2 ± 12.0 years; age range, 34–66; Fitzpatrick skin types III–IV) with intractable facial hyperpigmentation such as laser toning-resistant PMP, melasma, and pigmented contact dermatitis were enrolled in this pilot study between December 2015 and May 2016. This study was approved by the institutional review board of Asan Medical Center, Seoul, Korea. Written informed consent was obtained from all patients. 

We performed manual intradermal injection of Placentex^®^ at a dose of 0.05–0.1 mL per 1-cm^2^ injection site. A total average of 1 mL Placentex^®^ was injected in each subject per session. Almost all hyperpigmented areas received injections at 4-week intervals for a total of three sessions. The evaluations were conducted at baseline and at 4 weeks after these three treatment sessions. During evaluations, digital photographs were captured under identical conditions (i.e., room, light source, and camera settings) to document progress of patients.

Two independent dermatologists evaluated the digital photographs of patients using a 5-point scale as follows: 1, little or no improvement (0%–10%); 2, noticeable improvement (10%–25%); 3, fair improvement (25%–50%); 4, good improvement (50%–75%); and 5, excellent improvement (>75%).

### 4.7. Statistical Analysis 

The statistical significance of differences between the groups was assessed by analysis of variance (ANOVA) followed by Student’s *t* test. *p* values < 0.05 were considered significant.

## Figures and Tables

**Table 1:** Summary of clinical features and improvement scores of six patients with hyperpigmentation. PMP, photoaging-associated mottled pigmentation. F: female.

Patient Number	Sex	Age (Years)	Diagnosis	Area	Improvement Score
1	F	36	Melasma	Periocular	4
2	F	43	Melasma	Periocular	3
3	F	52	Melasma	Malar, cheek	5
4	F	34	PMP	Cheek	2
5	F	40	PMP	Cheek	2
6	F	66	Pigmented contact dermatitis	Face and neck	5
mean		45.2	-	-	3.7

## Abbreviations

AMP	adenosine monophosphate
ANOVA	analysis of variance
CT	Cholera toxin
DMEM	Dulbecco’s modified Eagle’s medium
DUSP	Dual specific phosphatase
EGF	Epidermal growth factor
ERK	Extracellular signal-regulated protein kinase
FBS	Fetal bovine serum
GSK3β	Glycogen synthase kinase 3β
HKGS	Human keratinocyte growth supplement
JNK	C-Jun N-terminal kinase
l-DOPA	l-3,4-dihydroxyphenylalanine
LPS	Lipopolysaccharide
MAP	Mitogen-activated protein
MITF	Microphthalmia-associated transcription factor
OD	Optical density
PBS	Phosphate-buffered saline
PDRN	Polydeoxyribonucleotide
PG	prostaglandin
PIH	Post-inflammatory hyperpigmentation
PI3K	Phosphatidylinositide 3-kinase
PMP	Photoaging-associated mottled pigmentation
PTU	*N*’-phenylthiourea
TNF	Tumor necrosis factor
TPA	Phorbol 12-myristate 13-acetate
TRP	Tyrosinase-related protein
UV	Ultraviolet
VEGF	Vascular endothelial growth factor
